# Supplementary material for: Composites of Titanium–Molybdenum Mixed Oxides and Non-Traditional Carbon Materials: Innovative Supports for Platinum Electrocatalysts for Polymer Electrolyte Membrane Fuel Cells
Source: Nanomaterials (Basel). 2024 Jun 19;14(12):1053. doi: 10.3390/nano14121053 (PMC11206414; doi:10.3390/nano14121053)
Supplement: Supplementary file 1 [file nanomaterials-14-01053-s001.zip › nanomaterials-3018939-supplementary.pdf]

SUPPLEMENTARY MATERIALS

Composites of Titanium–Molybdenum Mixed Oxides and Non-Traditional Carbon Materials: Innovative Supports for Platinum Electrocatalysts for Polymer Electrolyte Membrane Fuel Cells

Ilgar Ayyubov [1,2], Emília Tálas [1,*], Irina Borbáth [1], Zoltán Pászti [1], Cristina Silva [1,2], Ágnes Szegedi [1], Andrei Kuncser [3], M. Suha Yazici [4], István E. Sajó [5], Tamás Szabó [6,*] and András Tompos[1]

1 Institute of Materials and Environmental Chemistry, HUN-REN Research Centre for Natural Sciences, Magyar Tudósok körútja 2, H-1117 Budapest, Hungary; ilgar.ayyubov@ttk.hu (I.A.); borbath.irina@ttk.hu (I.B.); paszti.zoltan@ttk.hu (Z.P.); silva.cristina@ttk.hu (C.S.); szegedi.agnes@ttk.hu (Á.S.); tompos.andras@ttk.hu (A.T.)
2 Department of Physical Chemistry and Materials Science, Faculty of Chemical Technology and Biotechnology, Budapest University of Technology and Economics, Műegyetem rkp. 3., H-1111 Budapest, Hungary
3 National Institute of Materials Physics, 405A Atomistilor Street, 077125 Magurele, Romania; andrei.kuncser@infim.ro
4 Energy Institute, Istanbul Technical University, Maslak, 34467 Istanbul, Turkey; syazici@itu.edu.tr
5 Szentágothai Research Centre, University of Pécs, Ifjúság u. 20., H-7624 Pécs, Hungary; istvan.sajo@gmail.com
6 Department of Physical Chemistry and Materials Science, University of Szeged, Rerrich Béla tér 1, H-6720 Szeged, Hungary
* Correspondence: talas.emilia@ttk.hu (E.T.); sztamas@chem.u-szeged.hu (T.S.); Tel.: +36-1-3826-916 (E.T.); +36-62-544-112 (T.S.)

S1. Sample preparation

S1.1. Preparation of composite type of electrocatalyst supports

The preparation of $Ti_{(1-x)}Mo_xO_2$-C composite electrocatalyst supports was carried out in three separate steps: i) formation and aging of TiO_2 nuclei on the carbonaceous material, ii) addition of Mo-precursor and drying at low temperature, iii) high temperature heat treatment for Mo incorporation (see flow chart in Figure S1).

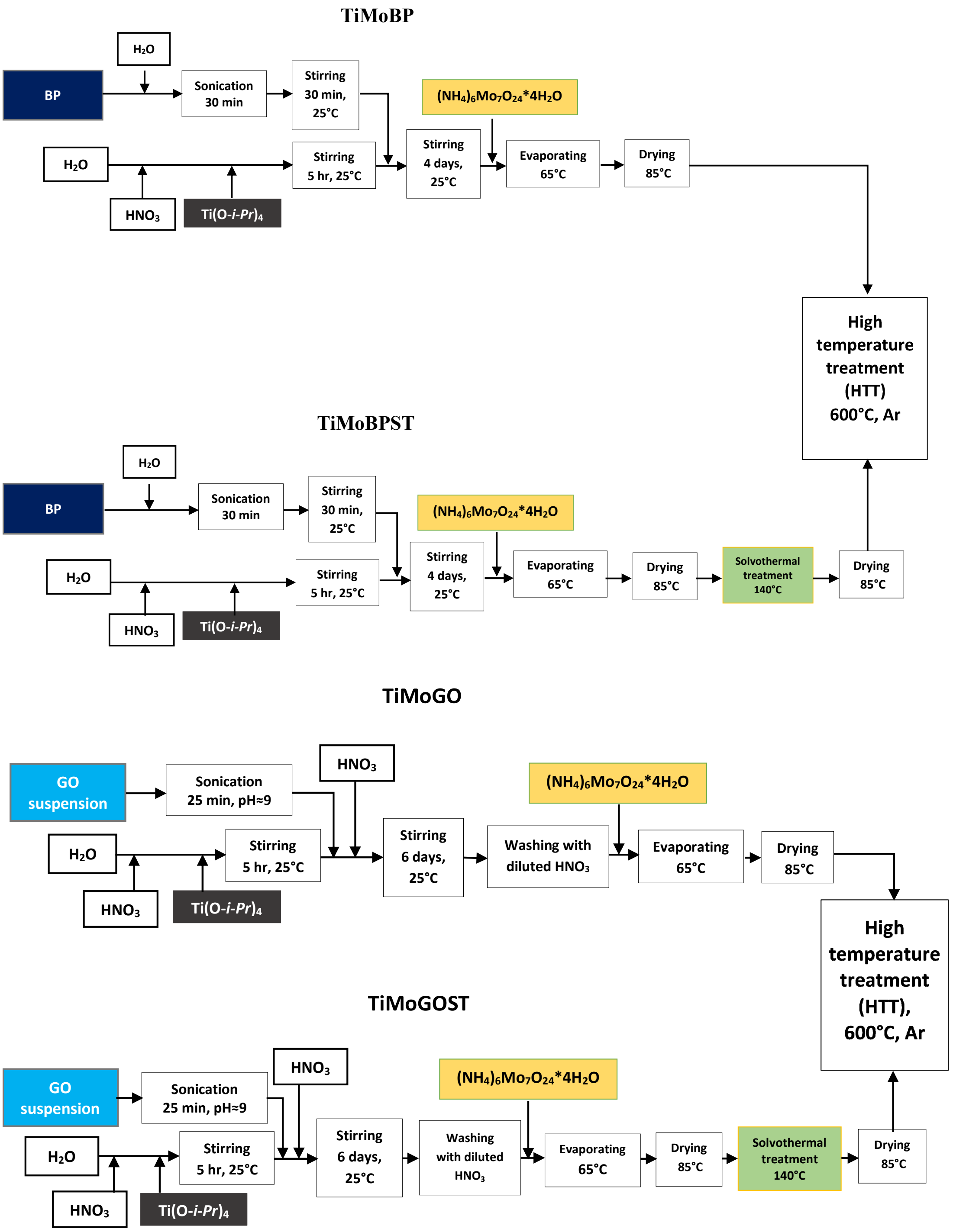

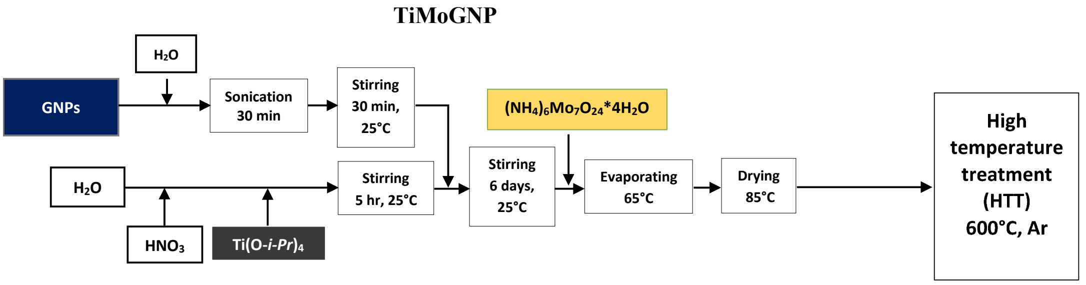

Figure S1. Flow charts for preparation of $Ti_{(1-x)}Mo_xO_2$-C composite electrocatalyst supports by using sol–gel-based multistep synthesis routes from different carbonaceous materials. BP: Black Pearls 2000 (Cabot); GO: graphite oxide, GNP: graphene nanoplatelets (Nanografi). ST: solvothermal treatment.

Typically, a transparent acidic TiO_2 colloidal solution was made first by adding Ti(O-*i*-*Pr*)$_4$ into the vigorously stirred mixture of cc. HNO_3 and distilled water. The acidic media provide rutile TiO_2 nuclei, which are necessary to the formation of mixed oxide in rutile phase in the final composite. In case of GO and GNP derived samples the volumes of H_2O and cc. HNO_3 were calculated to adjust the concentration of Ti(O-i-Pr)$_4$ to 0.115 M and of HNO_3 to 0.69 M in the first step of the procedure (5 h stirring). The aqueous suspension with the desired amount of GO or the amount of GNP was weighed. For exfoliation of GO, NaOH solution (pH~ 14) was added to the GO suspension till its pH became ~9. This suspension was sonicated for 25 min. Then the colloidal TiO_2 solution was poured to the GO suspension quickly under vigorous stirring. After that aqueous HNO_3 solution (prepared from 0.4 ml cc. HNO_3 and 7.9 ml H_2O) was added into the slurry. The pH of the slurry was adjusted to that of TiO_2 sol with cc. HNO_3 and the reaction mixture was stirred continuously for 6 days at room temperature for aging in order to facilitate rutile nuclei formation. After six days of aging, the mixture was centrifuged. The solid part was washed three times with diluted nitric acid in order to remove the well soluble $NaNO_3$. Finally, the solid was re-suspended in diluted HNO_3 of the same volume. After the removal of $NaNO_3$, the molybdenum precursor compound [$(NH_4)_6Mo_7O_{24}\times 4H_2O$] was added into the acidic slurry and it was stirred for 2 h at room temperature. Then the slurry was evaporated at 65 °C and dried overnight in the oven at 85 °C. By use of BP and GNP carbon source alkaline delamination and $NaNO_3$ removal was not needed. However, in case of GNP a very intensive ultrasonic treatment with Hielscher UP200S ultrasonic device was used.

In case of the solvothermal treatment step (ST), the solid material dried at 85 °C was sonicated in 100 ml 2-propanol in a short time (5-10 min) in an ultrasonic bath then the slurry was placed into a glass lined autoclave. After a nitrogen purge, the temperature inside was increased up to 140-150 °C in ~1 - 1.5 h and it was kept at this temperature for ~3 h on autogenic pressure. The reaction mixture was allowed to cool, and then it was centrifuged, air-dried at RT and dried again in an oven at 85 °C overnight as we described in our previous work in case of the TiO_2-GO derived carbon composite support [1].

As a final step of the synthesis of the catalyst support material, HTT at 600 °C in Ar atmosphere was performed for the molybdenum incorporation as described before [2].

S1.2. Synthesis of Pt electrocatalysts

$Ti_{(1-x)}Mo_xO_2$-C support materials were loaded with 20 wt.% Pt via a modified, sodium borohydride ($NaBH_4$) assisted ethylene-glycol (EG) reduction-precipitation method in order to obtain platinum containing electrocatalyst as we described before [2]. The flow chart of Pt loading process is depicted in Figure S2.

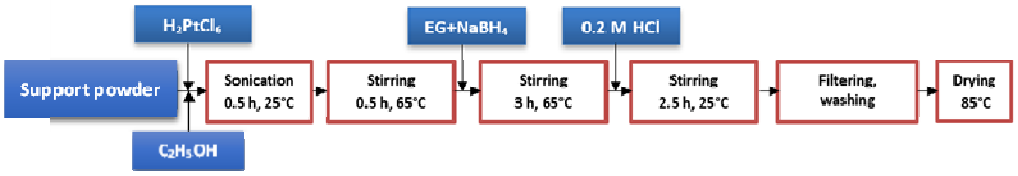

Figure S2. Flow chart of platinum loading.

S2. Characterization methods

S2.1. Physicochemical characterization methods

X-ray powder diffraction (XRD) patterns were obtained in a Philips model PW 3710 based PW 1050 Bragg-Brentano parafocusing goniometer using CuKα radiation (λ= 0.15418 nm), graphite monochromator and proportional counter. Silicon powder (NIST SRM 640) was used as an internal standard and the scans were evaluated with profile fitting methods. The cell parameters of the crystalline phases were determined from the fitted values.

Nitrogen physisorption measurements were carried out at temperature of liquid nitrogen using Thermo Scientific Surfer automatic volumetric adsorption analyzer (Thermo Fischer Scientific, Berlin, Germany). The specific surface area was calculated by the BET method in the range of relative pressures from 0.05 to 0.30.

Transmission Electron Microscopy (TEM) studies of the samples were made by use of a JEOL 3010 high resolution transmission electron microscope operating at 300 kV and by a Philips CM-10 transmission electron microscope with an accelerating voltage of 100 kV.

Scanning electron micrographs of the samples were recorded with a scanning electron microscope Vega II LMU model from Tescan, equipped with a spectrometer of energy dispersion of X-ray (EDX) Bruker Quantax 200, at the following operational parameters: accelerating voltage 30 kV, measuring time 1200 s, working distance around 17 mm, counting rate 0.4 kcps.

X-ray photoelectron spectroscopy (XPS) measurements were performed using an EA 125 electron spectrometer manufactured by OMICRON Nanotechnology GmbH (Germany). Ti, Mo and Pt content of the samples was measured by inductively coupled plasma-optical emission spectrometry (ICP-OES) technique by use of a simultaneous SPECTRO GENESIS instrument with axial plasma observation. Samples were measured after microwave assisted dissolution in 1:2:6 mixtures of concentrated hydrofluoric acid: hydrochloric acid: nitric acid.

The simultaneous thermogravimetric (TG) and mass spectrometric (MS) evolved gas analyses were recorded on a Setaram LabsysEvo (Setaram, Lyon, France) thermal analyzer, in high purity (99.9999%) He, with a flow rate of 90 cm^3/min. The measurements were done with a heating rate of 20 °C/min, in the temperature range of 25–1000 °C. Samples were weighed without any sample preparation into 100 μL alumina crucibles. The results were baseline-corrected, and then evaluated with the thermal analyzer's processing software (AKTS Calisto Processing, ver. 2.15). Parallel with the TG measurements, the analysis of the evolved gas was carried out on a Pfeiffer Vacuum OmniStar™ (Pfeiffer Vacuum, Asslar, Germany) gas analysis system. The gas splitter and transfer line to the mass spectrometer was preheated to 200 °C. The scanned m/z interval was 11–80 amu, with a scan speed of 50 ms amu-1. The mass spectrometer was operated in electron impact mode.

S2.2. Electrical conductivity measurements

Electrical current is conducted through powders by a percolating network of paths interrupted by series of interparticle tunnel junctions. Accordingly, in addition to the conductivity of the individual particles, a key contribution to the conductivity of the system arises from properties of these junctions. While intrinsic factors such as morphology or surface chemistry of the particles has obvious influence on the conductive nature of the junctions, extrinsic factors like their width and density, depending on the packing, alignment and geometry of the particles are equally important. As a result, the consistent understanding of the conductivity of powdered materials, involving the effects of external forces like compression, is very difficult to achieve [3–7]. At the same time, conductivity is very important in a range of applications, so the literature contains many efforts for its measurement [8–10]. Considering the range of factors influencing the conductivity, the results are very sensitive for the measurement conditions and even their interlaboratory comparison can be difficult [11]. However, the experience suggests that ranking of different materials according to their conductivity can be achieved by relatively simple methods. One of the quickest and simplest ways is a two-probe arrangement, in which the powder is controllably pressed against a static probe by a piston-like moving probe.

Electrical conductivity (σ) was estimated at room temperature, by means of impedance spectroscopy using a home-made equipment utilizing the two-probe principle (Figure S3).

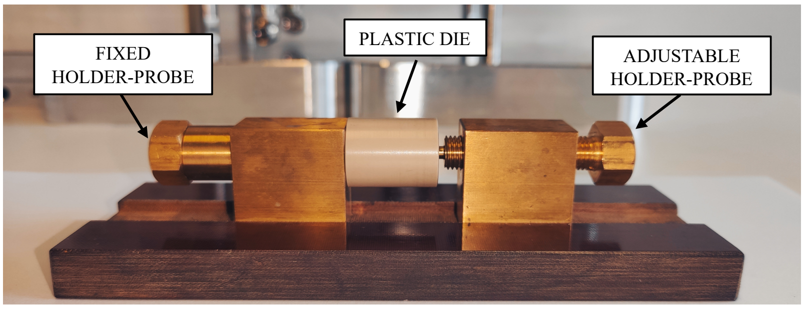

Figure S3. Setup for conductivity measurements.

The device consists of a cylindrical plastic tube and two brass holder-probes. The holders' tips are constructed as punches that slide within the tube (inner diameter: 3 mm), and their rear is threaded. The first punch's position is fixed while the other can be moved by turning its nut-shaped end by a wrench After inserting the powder sample, the adjustable punch was inserted, and the needed torque was applied with a torque wrench. The main advantages of this instrument are: simple and progressive compression of the sample powder, constraining of the particles within the die with constant mass and known dimensions, and simple disposal of the sample allowing reusability of the material, quick installation and easy cleaning.

A set of preliminary experiments was performed with Vulcan XC-72 and Black Pearls 2000 to assess the optimal working parameters of the device. Material powders were evaluated in amounts of 3, 5, and 7 mg, with 0.5, 1, and 1.5 Nm torque applied to each quantity. To test the compressibility of the probes, the same torque magnitudes were applied to empty sample probe-holders. At the maximum torque of 1.5 N m applied, the two-probe was compressed by a maximum of 0.33 mm. The volume fraction Φ (packing fraction) of particles is calculated for each powdered material and each load applied to the material column using the following Eq. S1:

$$\Phi = \frac{d}{d_{gr}} = \frac{m}{\pi r^2 h d_{gr}} \qquad \text{Eq. S1}$$

where h indicates the column height, r is the inner radius of the column ($r = 1.5$ mm), d is the apparent density of the powder column, d_{gr} is the absolute density of the substance (d_{gr} = ~1.8 g cm^{-3} for both materials), and m is the mass of material. In Figure S4, the material packing fraction is displayed versus the torque applied for both the Vulcan XC-72 (Figure S4A) and Black Pearls 2000 (Figure S4B). As the figures show, increasing the amount of applied compression increases the packing fraction linearly. When 7 mg of materials are tested, the R-squared values of the fitting lines are the greatest (Figure S4C), implying that the measurement accuracy of 7 mg powder is the highest.

The conductivity of the pressed materials was investigated in the potentiostatic electrochemical impedance spectroscopy mode of the Biologic SP 150 potentiostat in the 1 mHz-10 kHz range. Nyquist impedance curve of the powder samples is not half circle, but straight line obtained in IV quadrant. The resistive impedance is changing in a very limited range through the whole chosen frequency window. Since the measured reactance i.e., imaginary part of the impedance, is orders of magnitude smaller than resistance i.e., real part of the impedance, AC resistance is assumed to be equal to DC resistance at zero Hertz

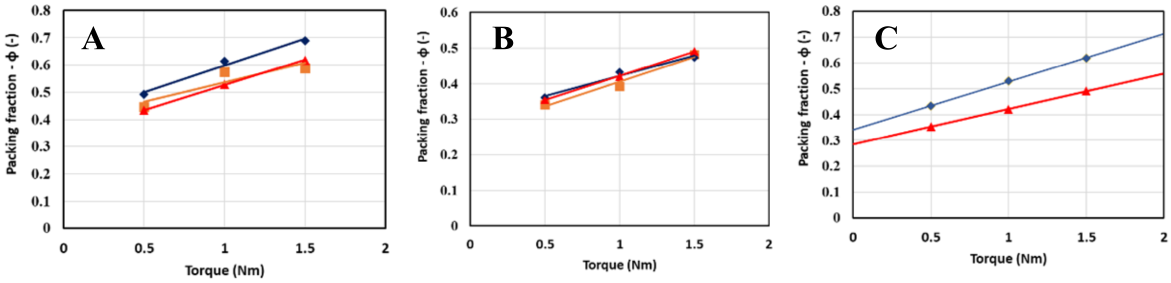

Figure S4. Packing fraction of Vulcan XC-72 (A) and Black Pearls 2000 (B) as a function of torque, when 0.5, 1, 1.5 Nm torque has been applied on 3 mg (square: ■), 5 mg (diamond: ♦), and 7 mg (triangle: ▲) material powder. Packing fraction of Vulcan XC-72 (diamond: ♦) and Black Pearls 2000 (triangle: ▲) as a function of torque (C), when 0.5, 1, 1.5 Nm torque has been applied on 7 mg material powder.

In order to obtain AC resistance at zero Hertz, the intercept of the fitting line of Re (Z) – f curve was determined where Re (Z) is resistance and f is the frequency. The conductivities of Vulcan XC-72 and Black Pearls 2000 are calculated via the Eq. S2:

$$\sigma = \frac{1}{R}\frac{h}{A} \qquad \text{Eq. S2}$$

Where R denotes the electrical resistance of the powder column, h the column height, and A the cross-sectional area of the column. After the conductivities are calculated, the σ – τ function of the measurements are plotted in Figure S5. The conductivities of the materials rise as the torque applied increases. With more density and fewer voids, there are more available conduction paths, and the resulting electrical conductivity is higher [12].

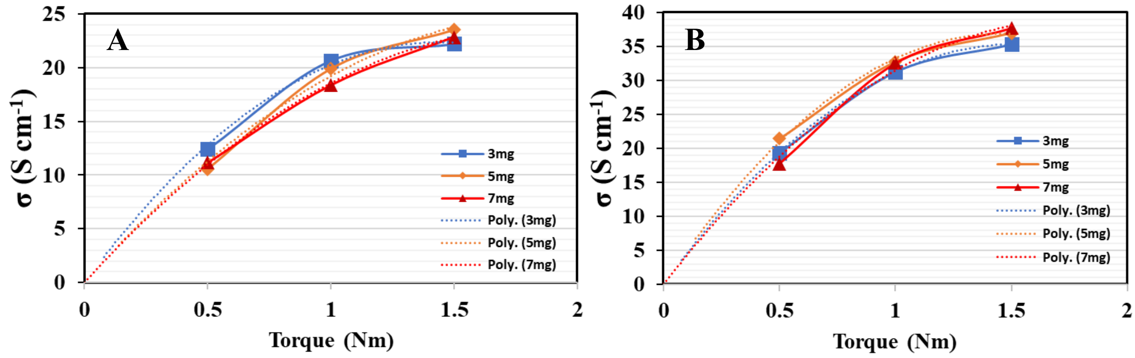

Figure S5. Electrical conductivities of Vulcan XC-72 (diamond: ♦) and Black Pearls 2000 (triangle: ▲) as a function of torque when 0.5, 1, 1.5 Nm torque has been applied on 3 mg (square: ■), 5 mg (diamond: ♦), and 7 mg (triangle: ▲) material powder, with fitting lines (dotted lines).

The conductivity values of the Vulcan XC-72 and Black Pearls 2000 are given in Table S1; the important observation is that the trend known from the literature ($\sigma_{Vulcan} > \sigma_{BP\ 2000}$) is correctly reproduced at all torques. At the same time, comparison of packing fraction/density and conductivity data with those presented in the literature [13] suggests that our instrument works at a relatively high level of compression, resulting in rather high conductivities. However, extrapolation of the σ – τ function to 0.1 Nm gives conductivity values comparable with the literature. Moreover, the conductivity ratios for the two carbon materials in the low torque limit (0.1-0.5 Nm) are in agreement with literature data. As a result, we may consider the measurement technique and the prepared two-probe apparatus to be accurate enough.

Table S1. Electrical conductivities of Vulcan XC-72 and Black Pearls 2000 when 0.5, 1, 1.5 Nm torque has been applied on 3 mg, 5 mg, and 7 mg material powder. Experimental values are the values calculated directly from data obtained by impedance spectroscopy. Extrapolation values are obtained via the equation of **σ – τ** function given in Figure S5. Literature values are obtained from the reference [3].

		**Literature**	**Extrapolation**	**Experimental**	**Experimental**	**Experimental**
		**n.d.**	**0.1 Nm**	**0.5 Nm**	**1 Nm**	**1.5 Nm**
**Material**	**Mass**	**σ[1] (S cm^{-1})**	**σ (S cm^{-1})**	**σ (S cm^{-1})**	**σ (S cm^{-1})**	**σ (S cm^{-1})**
**Vulcan XC-72**	3 mg	4.50	4.49	19.32	31.23	35.30
	5 mg	4.50	4.88	21.44	32.72	36.96
	7mg	4.50	4.25	17.72	32.54	37.70
**BP 2000**	3 mg	2.20	2.99	12.41	20.65	22.19
	5 mg	2.20	2.53	10.58	19.91	23.53
	7 mg	2.20	2.46	11.16	18.37	22.84

[1] amount of material is approximately 1 g.

S2.3. Electrochemical characterization

Electrochemically active Pt surface area (ECSA) was determined from the charge needed for oxidation of underpotentially deposited hydrogen on the platinum surface according to the traditional method. ECSA, *i.e.*, the area of the platinum surface on which electrochemical reactions may occur can be determined from CVs in the so-called hydrogen underpotential deposition region, between 0-350 mV, according to Eq. S3 [14,15].

$$ECSA_{Hupd}\,(cm^2) = Q_{ox_{Hupd}}\,(\mu C)\,/\,210\,(\mu C/cm^2) \qquad \text{Eq. S3}$$

where $Q_{ox_{Hupd}}$ is the charge accompanies oxidation of underpotentially deposited hydrogen, while 210 μC is the transported charge, while monolayer of H atoms is oxidized over 1 cm^2 Pt surface.

Electrocatalytic performance of the 20 wt.% Pt/$Ti_{0.8}Mo_{0.2}O_2$-C electrocatalysts was studied by cyclic voltammetry and CO_{ads}-stripping voltammetry measurements combined with stability test involving 500 polarization cycles and the second CO_{ads}-stripping voltammetry measurement. In the long-term stability test, the samples were submitted to cyclic polarization at a 100 mV s^{-1} scan rate for 10,000 cycles between 50 and 1000 mV potential limits; these measurements took ca. 54 hours.

The loss in electrochemically active Pt surface area upon N-cycle stability test ($\Delta ECSA_N$; N: 500, 2,500, 5,000 and 10,000) was calculated from the charges originated from the hydrogen desorption in the 1st and Nth cycles according to the Eq. S4:

$$\Delta ECSA_N = \{1-(ECSA_N/ECSA_1)\}\times 100\% \qquad \text{Eq. S4}$$

After every stability test, the electrolyte was changed to fresh one to avoid the re-deposition of the dissolved metals.

Catalytic activity of the catalyst samples was tested in the ORR by rotating disc electrode (RDE) technique. A RDE is a glassy carbon working electrode used in a three-electrode system. The rotating speed of the electrode can be controlled, yielding variable diffusion rate of the reactant. The ORR measurements were done in O_2 saturated 0.5 M H_2SO_4 solution. The diameter of RDE electrode and Pt loading used in these experiments was the same as during CV measurements. Polarization curves were recorded by cathodic scan sweeping the potential between 1000 and 200 mV with 10 mVs^{-1} sweep rate, rotating the electrode at 225, 400, 625, 900, 1225 and 1600 revolutions/min (rpm). In order to characterize the electrochemically active Pt surface area of the catalysts before and after the RDE measurements, 10 CVs between 50 and 1000 mV potential window in Ar saturated electrolyte were also measured.

S3. Results

S3.1. Physisorption measurements

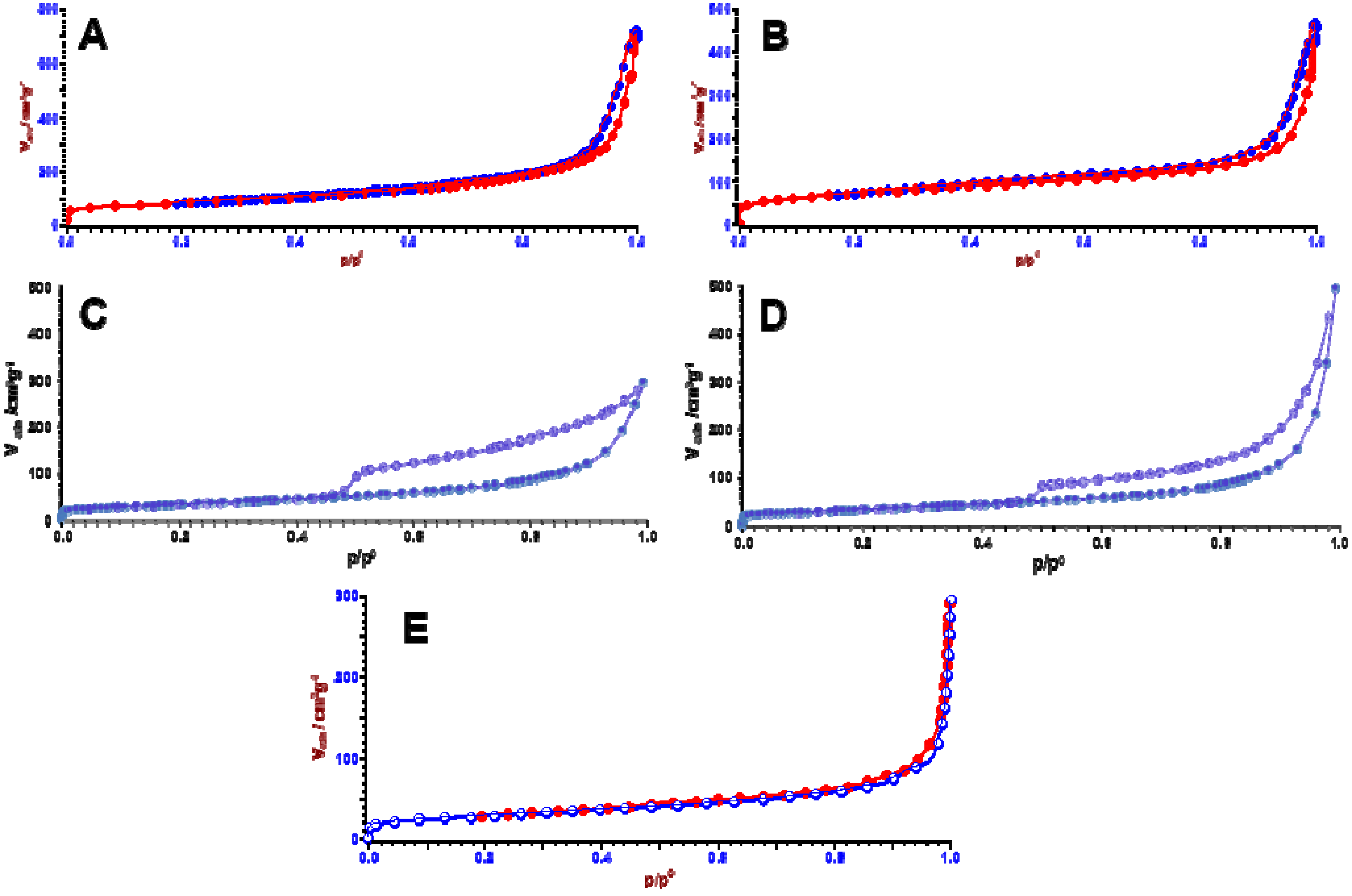

Figure S6. Adsorption isotherms of $Ti_{(x-1)}Mo_xO_2$-C composites. A: TiMoBP, B: TiMoBPST, C: TiMoGO, D: TiMoGOST, E: TiMoGNP.

S3.2. Thermal analysis

Even without a complete analysis, it can be seen that thermal changes of the selected TiMoGO composite can be divided into two main intervals, such as low temperature changes (range ~29-300 °C) and high temperature changes (range ~700-900 °C) both well delimitable in case of sample obtained after the drying step at 80°C (Fig. S7A) and sample obtained after the HTT step (600°C) (Fig. S7B).

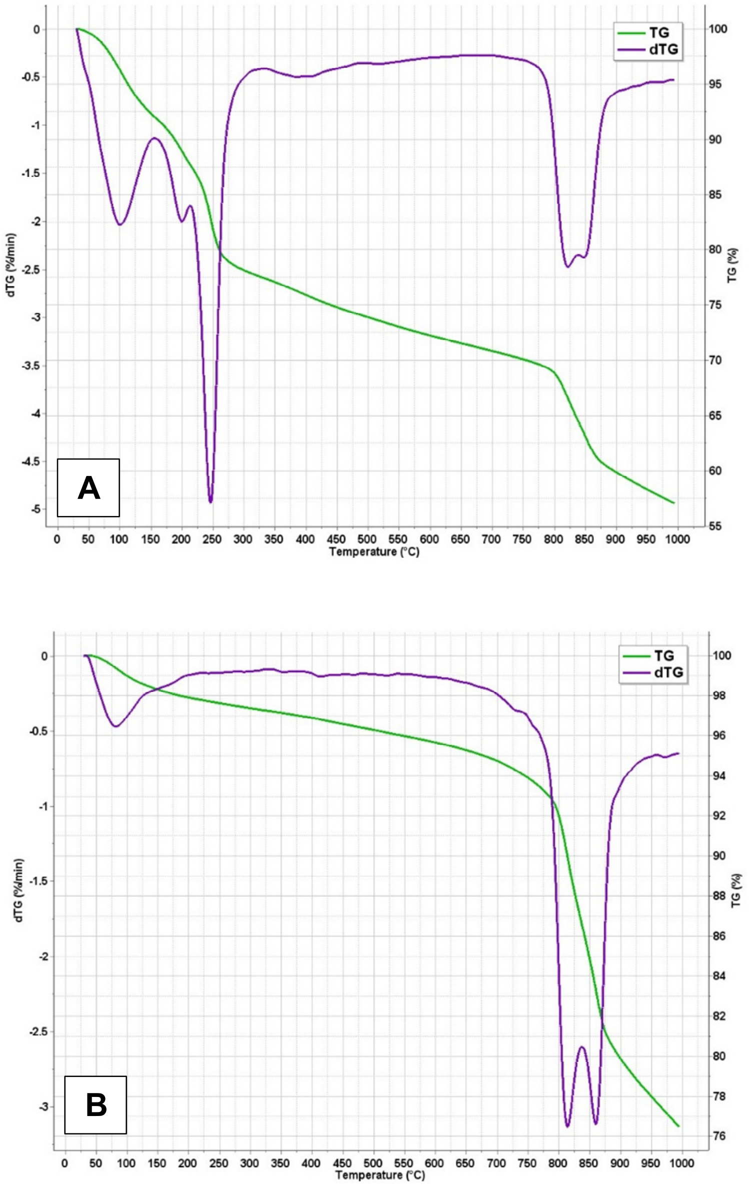

Figure S7. Mass (TG) and mass loss rate (DTG) curves of TiMoGO composite before high temperature heat treatment (A), after high temperature heat treatment (B).

Based on the TG-MS trace (Figure S8 column A) it can be concluded that low temperature region up to ~150°C is related to the removal of adsorbed water. Parallel formation H_2O, CO_2, CO, and formaldehyde (H_2CO) between ~150-300°C is due to the partly removal of the oxygen containing functional groups from the carbonaceous part of the composite in line with the literature finding of the decomposition of GO[16]. Ref. [16] summarized that oxygen-containing groups of GO were generally decomposed in three temperature regions of 170–250, 500–600, and 750–1000 C. In case of our, composite incorporated GO the decomposition in the middle region is hardly detectable, while the high-temperature region degradation is clearly visible. It should also be noted that in case of sample

after HTT (Figure S8 column B) the signal intensities were much lower and the low-temperature region had almost disappeared, obviously as a result of the preliminary heat treatment, only moisture desorption is present. All these observations indicate that during HTT, not only the mixed oxide part of the composite, but also its carbonaceous content undergoes significant changes.

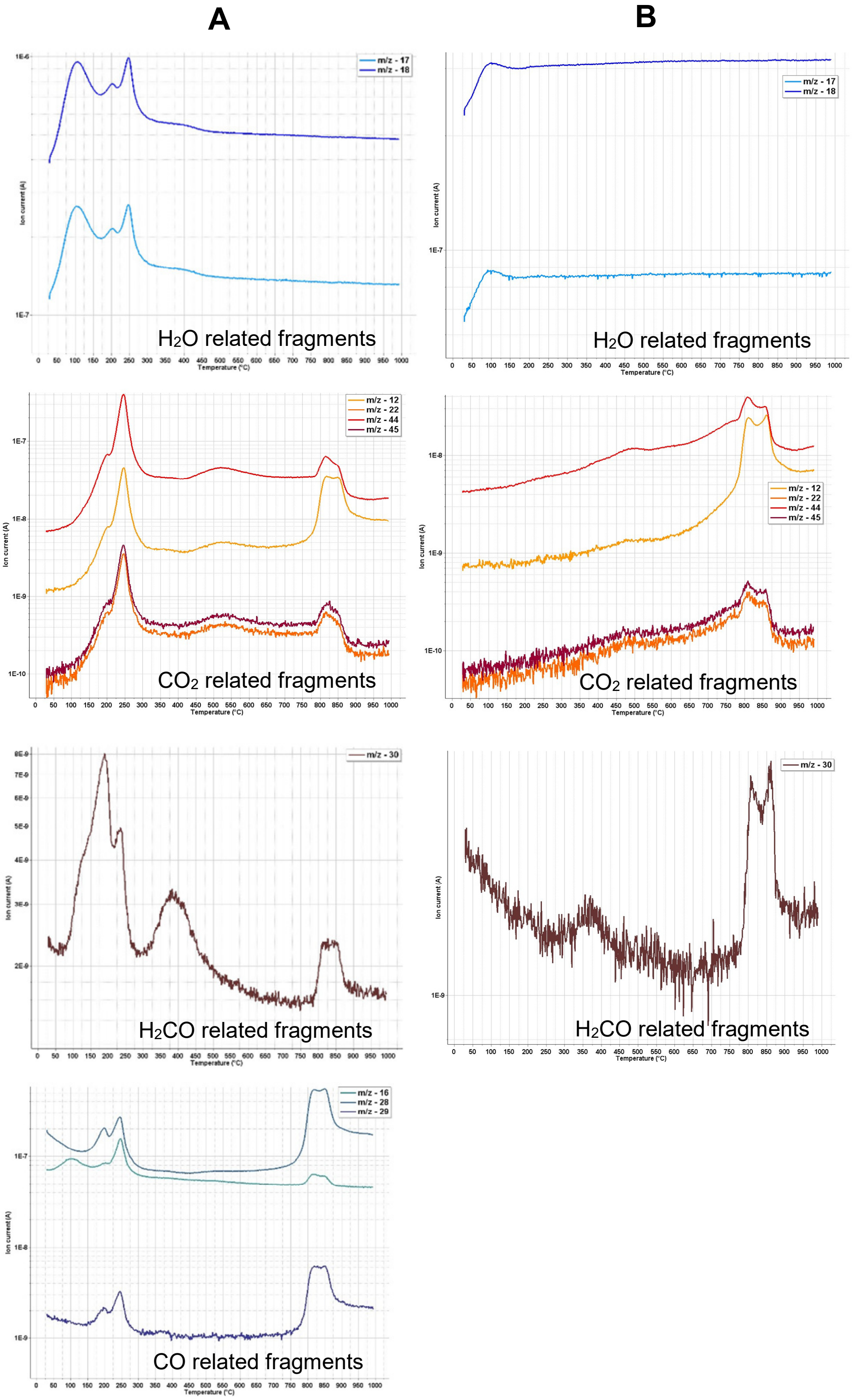

Figure S8. TG-MS trace of the of TiMoGO composite before high temperature heat treatment (column A), after high temperature heat treatment (column B).

S3.3. Characterization by XPS

The results of XPS measurements are presented in Figures S9-S11 and in Tables S2-S3.

Mo 3d spectra collected on the composite supports as well as on the Pt-loaded electrocatalyst samples are compared in Fig. S9. The measured data were evaluated as described previously [17], [18], [19]. The Mo^{6+} ionic state was modeled by a simple spin-orbit doublet with its $3d_{5/2}$ component around 232.5 eV binding energy; the $3d_{5/2}/3d_{3/2}$ intensity ratio (1:0.66) and doublet separation (3.1 eV) were based on reference spectra of a MoO_3 sample. Mo^{5+} was described by a similar spin-orbit doublet with the $3d_{5/2}$ component around 231 eV. The Mo^{4+} contribution was represented by a complex line shape arising from coexistence of differently screened final states, with the leading peak around 230 eV; the line shape was derived from measurements on MoO_2.

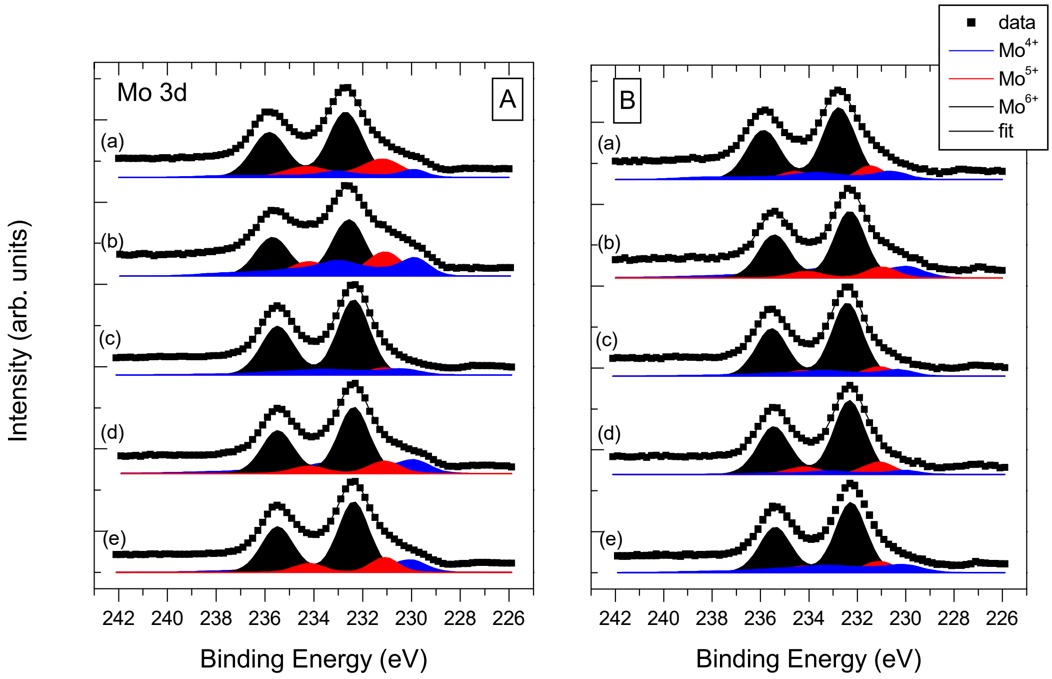

Figure S9. Mo 3d core level spectra of (A): the investigated $Ti_{(1-x)}Mo_xO_2$-C composite supports and (B) the corresponding electrocatalysts. Spectra are identified according to the support: (a): TiMoGO, (b): TiMoGoST, (c): TiMoBP, (d): TiMoBPST, (e): TiMoGNP.

The Mo 3d spectra of both the supports and the catalysts closely resembled those reported in our previous works on related systems [17–19]. The dominant Mo^{6+} contribution was always accompanied by weaker Mo^{5+} and Mo^{4+} signals. Nevertheless, some correlation existed between the choice of the carbonaceous component and/or the preparation route and the reduced Mo content (Mo^{5+}, Mo^{4+}) of the composite. The weakest amount of reduced Mo was found in the BP-based support, while the solvothermal step always tended to increase the Mo reduction level. However, after Pt deposition only marginal differences were found in the Mo 3d spectrum of the catalysts.

C 1s spectra of the composite supports and the related electrocatalysts are shown in Fig. S10. The measured spectra were processed by following routes described in a previous [20]. Accordingly, the C 1s spectra were approximated by an asymmetric line shape corresponding to graphite-like sp^2-bound carbon atoms measured on a highly graphitic reference sample (main peak at 284.4 eV binding energy), completed by small additional peaks arising from heteroatom-bound carbon species. The minor peak around 286 eV was attributed to C singly bound to O like in C-OH or C-O-C (epoxide, cyclic ether) groups, while the contribution around 288.5 eV is due to carboxylic groups [21,22].

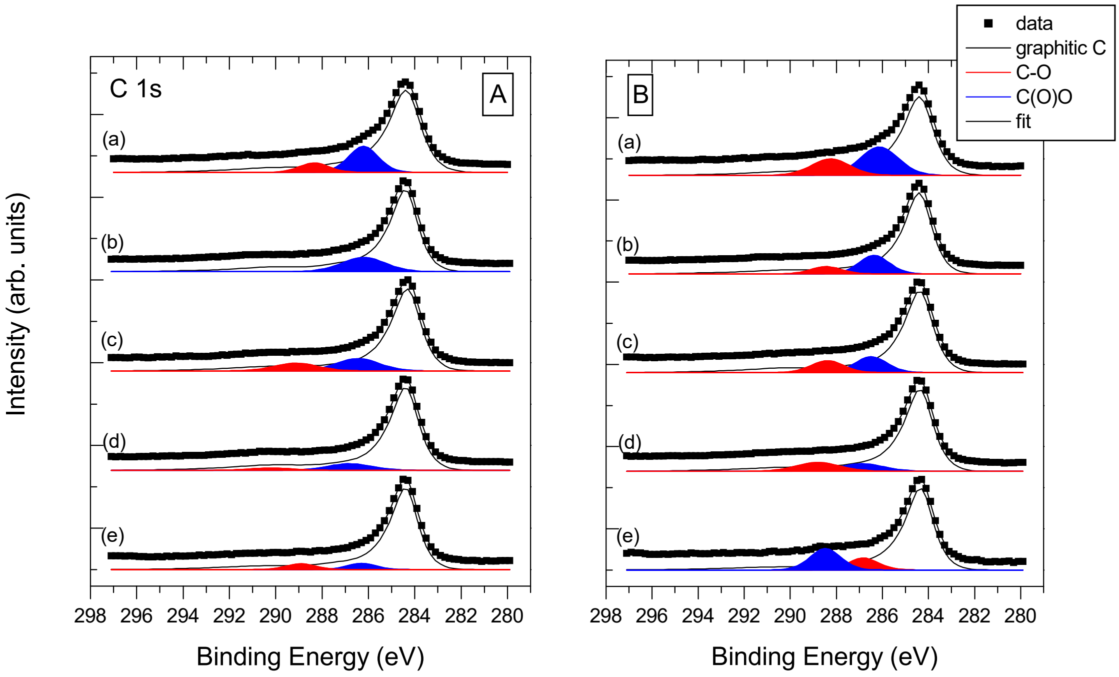

Figure S10. C 1s core level spectra of (A): the investigated $Ti_{(1-x)}Mo_xO_2$-C composite supports and (B) the corresponding electrocatalysts. Spectra are identified according to the support: (a): TiMoGO, (b): TiMoGoST, (c): TiMoBP, (d): TiMoBPST, (e): TiMoGNP. The heteroatom-related contributions are shown after multiplication by 5 for better visibility.

The C 1s spectra of all samples were dominated by the graphitic carbon contribution, demonstrating the predominantly graphitic nature of the carbonaceous backbones in both the composite supports and the electrocatalysts. Only the GO-based composite demonstrated a slightly higher functional group content as a remainder of the very highly functionalized parent graphite oxide structure. Nevertheless, the solvothermal treatment removed a significant fraction of these groups. The same effect was evident in case of the BP-based systems as well. As expected, the functional group content of the carbonaceous component of the GNP-based composite was minimal (Fig. S10A). After Pt loading the C 1s spectra of the catalysts supported on different composites was rather similar, indicating the presence of a small amount of oxidized carbon species in all cases.

In Figure S11A O 1s spectra of the composite supports are presented. The spectra are in all systems dominated by a peak around 530.0 eV binding energy, which can be attributed to lattice oxide of the Mo-doped titania component [22]. Additional small peaks around 531.5 eV are typically assigned to -OH groups on the metal oxide, but O atoms in epoxide or cyclic ether-like environments can also give a contribution to this binding energy range [21]. A higher binding energy O 1s contribution around 533 eV occurred with considerable intensity only in the spectra of the composites based on GO. Adsorbed moisture or carbon-bound hydroxyl, carboxyl groups can contribute to this peak [21,22]. The relatively intense high binding energy region of the O 1s spectrum of the TiMoGOST composite is somewhat surprising but can perhaps be related to the reduced nature of its oxide component (indicated by the higher Mo^{5+}, Mo^{4+} content), which may enhance its reactivity towards water. Pt loading caused only marginal changes in the O 1s spectra so they are not shown here.

The Pt 4f spectra of the electrocatalysts deposited on the composite supports are shown in Fig. S11B. The Pt content was mainly metallic in all cases (Pt $4f_{7/2}$ binding energy around 71.3 eV [22]); the small and apparently randomly varying ionic Pt contributions (Pt^{2+} at 72.6 eV, Pt^{4+} at 75 eV [22]) were attributed to slight surface oxidation caused by air exposure.

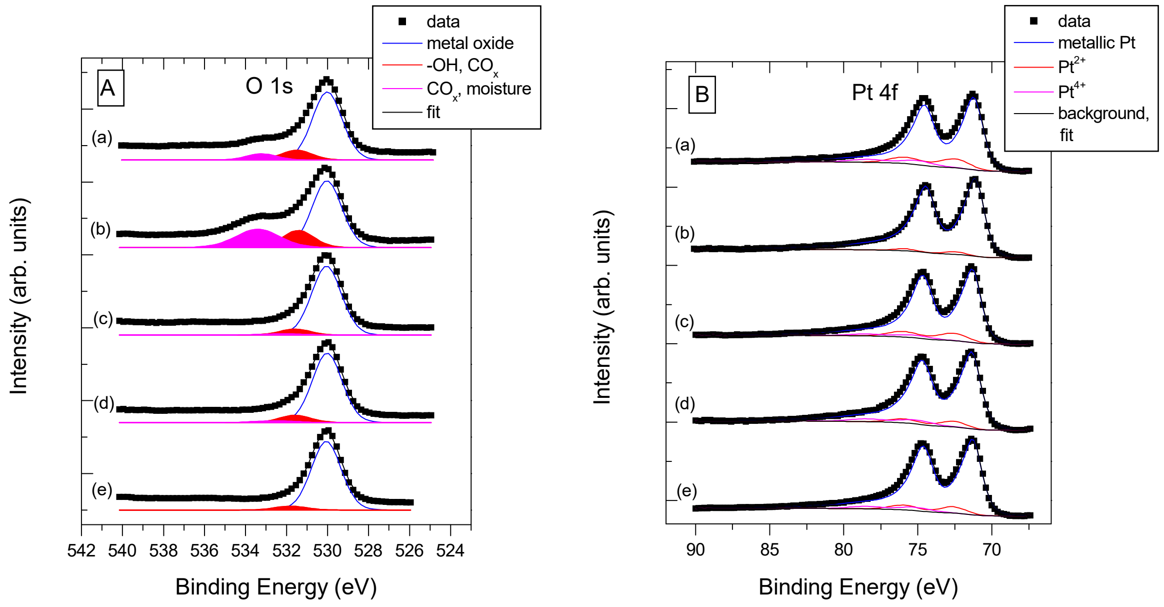

Figure S11. (A): O 1s core level spectra of the investigated $Ti_{(1-x)}Mo_xO_2$-C composite supports. (B) Pt 4f spectra of the investigated electrocatalysts. Spectra are identified according to the support: (a): TiMoGO, (b): TiMoGoST, (c): TiMoBP, (d): TiMoBPST, (e): TiMoGNP.

Table S2. XPS results of $Ti_{(1-x)}Mo_xO_2$-C [1] composites

Sample ID	Composition	Chemical state			
	(in at%)	BE [2] (ratio)	BE [2] assign.	BE [2] assign.	BE [2] assignment
		Mo 3d	Ti 2p	O 1s	C 1s
TiMoGO	Mo: 4.0<br>Ti: 6.4<br>O: 27.5<br>C: 62.1	Mo^{4+}: 229.6 (19%)<br>Mo^{5+}: 231.0 (20%)<br>Mo^{6+}: 232.3 (61%)	458.6 TiO_2	530.0 metal oxide<br>531.4 -OH [3](12%)<br>533.3 water [3] (8%)	284.4 graphitic C<br>286.1 C-O (4%)<br>288.2 C(O)-O (1%)
TiMoGOST	Mo: 1.7<br>Ti: 5.8<br>O: 25.2<br>C: 67.5	Mo^{4+}: 229.7 (30%)<br>Mo^{5+}: 231.0 (22%)<br>Mo^{6+}: 232.4 (48%)	458.7 TiO_2	530.0 metal oxide<br>531.3 -OH [3] (16%)<br>533.4 water [3] (24%)	284.4 graphitic C<br>286.1 C-O (3%)
TiMoBP	Mo: 3.5<br>Ti: 7.9<br>O: 27.6<br>C: 61.0	Mo^{4+}: 230.4 (14% [4]-20% [5])<br>Mo^{5+}: 231.3 (7% [4] -10% [5])<br>Mo^{6+}: 232.5 (79% [4] -70% [5])	458.7 TiO_2	530.0 metal oxide<br>531.5 -OH, CO_x (8%)<br>532.5 water [3] (2%)	284.4 graphitic C<br>286.5 C-O (3%)<br>288.5 C(O)-O (1%)
TiMoBPST	Mo: 2.7<br>Ti: 6.2<br>O: 20.9<br>C: 70.2	Mo^{4+}: 229.8 (27% [4] -28% [5])<br>Mo^{5+}: 231.3 (11% [4] -12% [5])<br>Mo^{6+}: 232.5 (62% [4] -60% [5])	458.6 TiO_2	530.0 metal oxide<br>531.6 -OH, CO_x (10%)<br>532.6 water [3] (3%)	284.4 graphitic C<br>286.5 C-O (1%)<br>288.5 C(O)-O (<1%)
TiMoGNP	Mo: 4.8<br>Ti: 14.5<br>O: 43.6<br>C: 37.1	Mo^{4+}: 229.9 (21% [4] -22% [5])<br>Mo^{5+}: 231.1 (12% [4] -18% [5])<br>Mo^{6+}: 232.5 (67% [4] -60% [5])	458.6 TiO_2	530.0 metal oxide<br>531.5 -OH, CO_x (6%)	284.4 graphitic carbon<br>286.3 C-O (1%)<br>288.9 C(O)-O (<1%))

[1] nominal Mo:Ti ratio: 20:80; nominal mixed oxide:carbonaceous material ratio:75:25.
[2] BE: binding energy (in eV).
[3] the O 1s component around 531 eV contains also contributions from epoxide groups on the carbon backbone and the peak around 533 eV contains also contributions from –OH and carboxylic groups bound to carbon.
[4] at the first measurement.
[5] after 3h X-ray exposition.

Table S3. XPS results of 20 wt.% Pt/$Ti_{(1-x)}Mo_xO_2$-C [1] catalysts.

**Sample ID**	**Composition**	**Chemical state**				
	**(in at%)**	**BE [2] (ratio)**	**BE [2] assign.**	**BE [2] assign.**	**BE [2] assign.**	**BE [2] (ratio)**
		**Mo 3d**	**Ti 2p**	**O 1s**	**C 1s**	
Pt/TiMoGO	Mo: 2.2<br>Ti: 5.4<br>O: 26.2<br>C: 60.2<br>Pt: 5.3	Mo^{4+}: 230.0 (16%)<br>Mo^{5+}: 231.1 (17%)<br>Mo^{6+}: 232.4 (67%)	458.6<br>TiO_2	530.1 metal oxide<br>531.6 -OH, C-O-C (18%)<br>533.3 carboxylic group, water (13%)	284.4 graphitic C<br>286.1 C-O (6%)<br>288.3 C(O)-O (3%)	71.2 Pt^0<br>72.5 Pt^{2+}: PtO, $Pt(OH)_2$<br>75.0 Pt^{4+}: PtO_2
Pt/TiMoGOST	Mo: 1.6<br>Ti: 4.3<br>O: 17.3<br>C: 70.3<br>Pt: 6.5	Mo^{4+}: 229.9 (22% [3]-31% [4])<br>Mo^{5+}: 231.1 (12% [3] -16% [4])<br>Mo^{6+}: 232.4 (66% [3] -53% [4])	458.6<br>TiO_2	530.0 metal oxide<br>531.5 -OH, C-O-C (17%)<br>533.2 carboxylic group, water (10%)	284.4 graphitic C<br>286.3 C- O (3%)<br>288.3 C(O)-O (1%)	71.2 Pt^0<br>72.6 Pt^{2+}: PtO, $Pt(OH)_2$<br>74.9 Pt^{4+}: PtO_2
Pt/TiMoBP	Mo: 2.0<br>Ti: 6.4<br>O: 24.5<br>C: 61.2<br>Pt: 5.9	Mo^{4+}: 230.3 (14% [3]-20% [4])<br>Mo^{5+}: 231.2 (9% [3] -14% [4])<br>Mo^{6+}: 232.5 (77% [3] -66% [4])	458.7<br>TiO_2	530.0 metal oxide<br>531.4 -OH, C-O-C (10%)<br>532.9 carboxylic group, water (3%)	284.4 graphitic C<br>286.3 C-O (3%)<br>288.4 C(O)-O (2%)	71.3 Pt^0<br>72.6 Pt^{2+}: PtO, $Pt(OH)_2$<br>75.1 Pt^{4+}: PtO_2
Pt/TiMoBPST	Mo: 2.0<br>Ti: 5.4<br>O: 22.1<br>C: 64.7<br>Pt: 5.8	Mo^{4+}: 230.2 (7%[c]-16% [4])<br>Mo^{5+}: 231.1 (13% [c] -12% [4])<br>Mo^{6+}: 232.4 (80% [c] -72% [4])	458.6<br>TiO_2	529.9 metal oxide<br>531.2 -OH, C-O-C (11%)<br>532.6 carboxylic group, water (4%)	284.4 graphitic C<br>286.7 C-O (2%)<br>288.7 C(O)-O (2%)	71.4 Pt^0<br>72.6 Pt^{2+}: PtO, $Pt(OH)_2$<br>75.1 Pt^{4+}: PtO_2
Pt/TiMoGNP	Mo: 3.7<br>Ti: 12.1<br>O: 38.5<br>C: 36.0<br>Pt: 9.7	Mo^{4+}: 230.0 (19% [3]-18% [4])<br>Mo^{5+}: 231.1 (11% [3]-15% [4])<br>Mo^{6+}: 232.4 (70% [3] -67% [4])	458.6<br>TiO_2	529.9 metal oxide<br>531.4 -OH, C-O-C (10%)	284.4 graphitic C<br>286.5 C-O (2%)<br>288.6 C(O)-O (4%)	71.3 Pt^0<br>72.6 Pt^{2+}: PtO, $Pt(OH)_2$<br>75.1 Pt^{4+}: PtO_2

[1] nominal Mo:Ti ratio: 20:80; nominal mixed oxide:carbonaceous material ratio: 75:25. [2] BE: binding energy (in eV)
[3] at the first measurement; [4] after 3h X-ray exposition.

S3.4. Elemental mapping

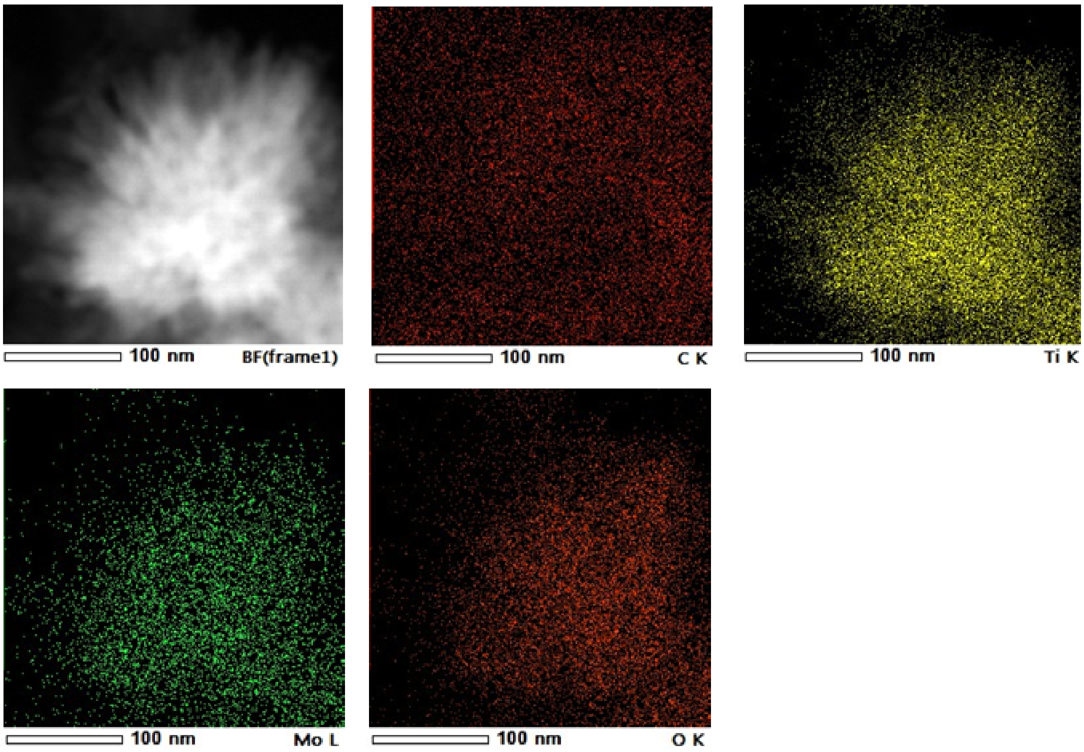

Figure S12. Chemical mapping images for the TiMoBP sample.

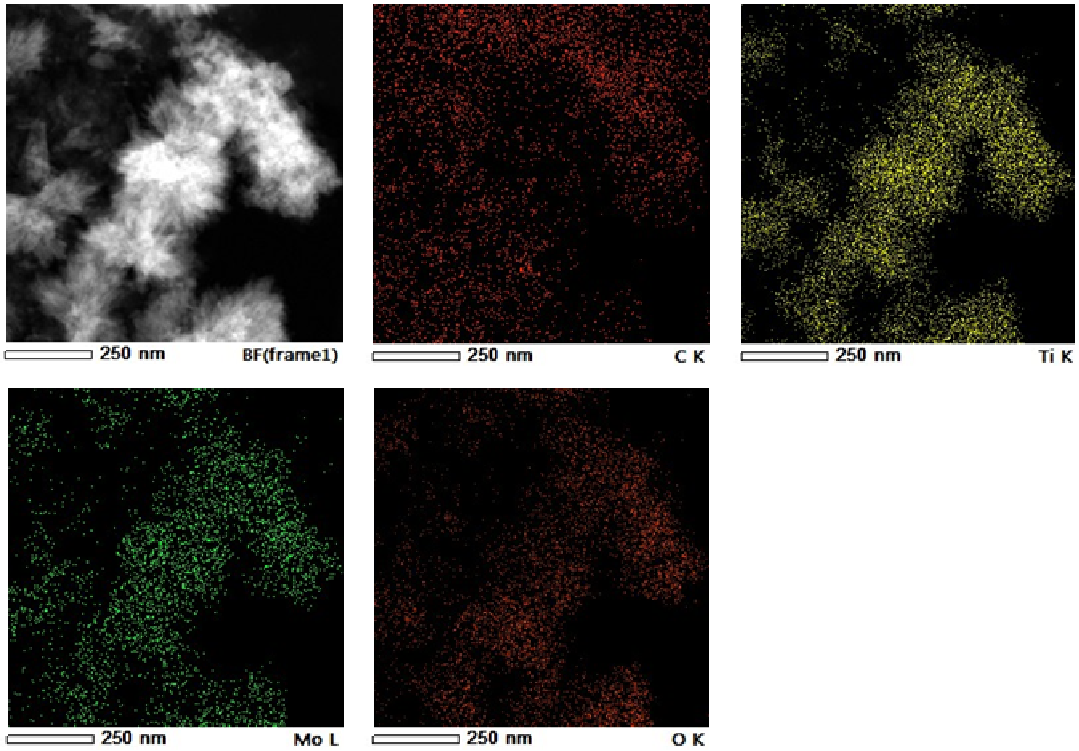

Figure S13. Chemical mapping images for the TiMoBPST sample.

S3.5. Electrochemical characterization

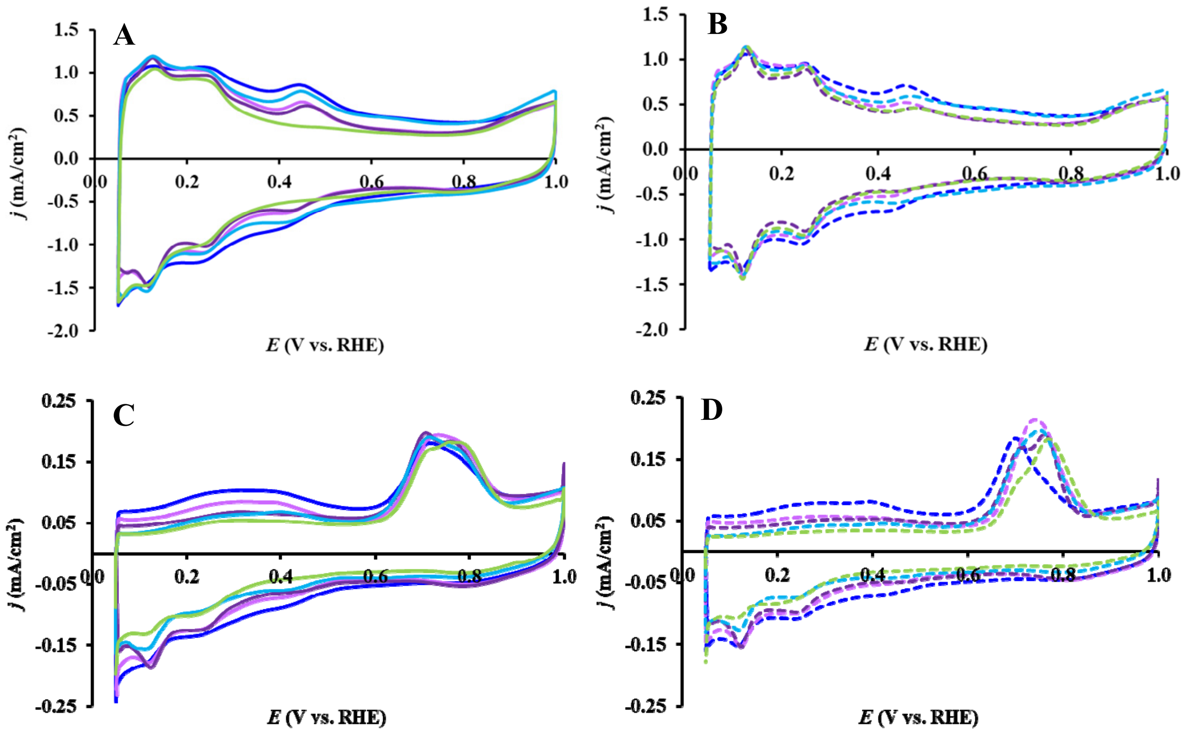

Figure S14. Cyclic voltammograms and CO_{ads} stripping voltammograms of the electrocatalysts recorded in 0.5 M H_2SO_4 before (A, C: solid curves) and after 500 cycles (B, D: dashed curves) of the stability test: Pt/TiMoGO (blue (■)), Pt/TiMoBP (light blue (■)), Pt/TiMoGOST (light violet (■)), Pt/TiMoBPST (violet (■)) and Pt/TiMoGNP (green (■)). Sweep rate: 100 mV/s (A, B) and 10 mV/s (C, D).

References

1. Ayyubov, I.; Borbáth, I.; Pászti, Z.; Sebestyén, Z.; Mihály, J.; Szabó, T.; Illés, E.; Domján, A.; Florea, M.; Radu, D.; et al. Synthesis and Characterization of Graphite Oxide Derived TiO_2-Carbon Composites as Potential Electrocatalyst Supports. *Top. Catal.* **2021**, doi:10.1007/s11244-021-01513-1.
2. Vass, Á.; Borbáth, I.; Pászti, Z.; Bakos, I.; Sajó, I.E.; Németh, P.; Tompos, A. Effect of Mo Incorporation on the Electrocatalytic Performance of Ti–Mo Mixed Oxide–Carbon Composite Supported Pt Electrocatalysts. *React. Kinet. Mech. Catal.* **2017**, *121*, 141–160, doi:10.1007/s11144-017-1155-5.
3. Pantea, D.; Darmstadt, H.; Kaliaguine, S.; Roy, C. Electrical Conductivity of Conductive Carbon Blacks: Influence of Surface Chemistry and Topology. *Appl. Surf. Sci.* **2003**, *217*, 181–193, doi:10.1016/S0169-4332(03)00550-6.
4. Montes, J.M.; Cuevas, F.G.; Ternero, F.; Astacio, R.; Caballero, E.S.; Cintas, J. A Method to Determine the Electrical Resistance of a Metallic Powder Mass under Compression. *Metals (Basel).* **2017**, *7*, 1–9, doi:10.3390/met7110479.
5. Celzard, A.; Marêché, J.F.; Payot, F.; Furdin, G. Electrical Conductivity of Carbonaceous Powders. *Carbon N. Y.* **2002**, *40*, 2801–2815, doi:10.1016/S0008-6223(02)00196-3.
6. Celzard, A.; Marêché, J.F.; Payot, F. Simple Method for Characterizing Synthetic Graphite Powders. **2000**, *33*, 1556–1563.
7. Pantea, D.; Darmstadt, H.; Kaliaguine, S.; Sümmchen, L.; Roy, C. Electrical Conductivity of Thermal Carbon Blacks: Influence of Surface Chemistry. *Carbon N. Y.* **2001**, *39*, 1147–1158, doi:10.1016/S0008-6223(00)00239-6.
8. Morris, D.R.P.; Liu, S.P.; Villegas Gonzalez, D.; Gostick, J.T. Effect of Water Sorption on the Electronic Conductivity of Porous Polymer Electrolyte Membrane Fuel Cell Catalyst Layers. *ACS Appl. Mater. Interfaces* **2014**, *6*, 18609–18618, doi:10.1021/am503509j.
9. Macías-García, A.; Díaz-Díez, M.A.; Alfaro-Domínguez, M.; Carrasco-Amador, J.P. Influence of Chemical

Composition, Porosity and Fractal Dimension on the Electrical Conductivity of Carbon Blacks. *Heliyon* **2020**, *6*, e04024, doi:10.1016/j.heliyon.2020.e04024.
10. Fraser, A.; Zhang, Z.; Merle, G.; Gostick, J.; Barralet, J. Powder Conductivity Assessment Using a Disposable 3D Printed Device. *Electroanalysis* **2018**, *30*, 1897–1901, doi:10.1002/elan.201800145.
11. Marsden, A.J.; Papageorgiou, D.G.; Vallés, C.; Liscio, A.; Palermo, V.; Bissett, M.A.; Young, R.J.; Kinloch, I.A. Electrical Percolation in Graphene-Polymer Composites. *2D Mater.* **2018**, *5*, doi:10.1088/2053-1583/aac055.
12. Khodabakhshi, S.; Fulvio, P.F.; Andreoli, E. Carbon Black Reborn: Structure and Chemistry for Renewable Energy Harnessing. *Carbon N. Y.* **2020**, *162*, 604–649, doi:10.1016/j.carbon.2020.02.058.
13. Sánchez-González, J.; MacÍas-García, A.; Alexandre-Franco, M.F.; Gómez-Serrano, V. Electrical Conductivity of Carbon Blacks under Compression. *Carbon N. Y.* **2005**, *43*, 741–747, doi:10.1016/j.carbon.2004.10.045.
14. Woods, R. *Electroanalytical Chemistry: A Series of Advances*; Bard, A.J., Ed.; M. Dekker: New York; Basel, 1976; Vol. 9, pp. 1–162;.
15. Schulenburg, H.; Durst, J.; Müller, E.; Wokaun, A.; Scherer, G.G. Real Surface Area Measurements of Pt_3Co/C Catalysts. *J. Electroanal. Chem.* **2010**, *642*, 52–60, doi:10.1016/j.jelechem.2010.02.005.
16. Gudkov, M. V.; Bazhenov, S.L.; Bekhli, L.S.; Mel'nikov, V.P. Explosive Reduction of Graphite Oxide. *Russ. J. Phys. Chem. B* **2018**, *12*, 860–868, doi:10.1134/S199079311805007X.
17. Vass, Á.; Borbáth, I.; Bakos, I.; Pászti, Z.; Sajó, I.E.; Tompos, A. Novel Pt Electrocatalysts: Multifunctional Composite Supports for Enhanced Corrosion Resistance and Improved CO Tolerance. *Top. Catal.* **2018**, *61*, 1300–1312, doi:10.1007/s11244-018-0988-0.
18. Diczházi, D.; Borbáth, I.; Bakos, I.; Szijjártó, G.P.; Tompos, A.; Pászti, Z. Design of Mo-Doped Mixed Oxide–Carbon Composite Supports for Pt-Based Electrocatalysts: The Nature of the Mo-Pt Interaction. *Catal. Today* **2021**, *366*, 31–40, doi:10.1016/j.cattod.2020.04.004.
19. Gubán, D.; Tompos, A.; Bakos, I.; Vass; Pászti, Z.; Szabó, E.G.; Sajó, I.E.; Borbáth, I. Preparation of CO-Tolerant Anode Electrocatalysts for Polymer Electrolyte Membrane Fuel Cells. *Int. J. Hydrogen Energy* **2017**, *42*, 13741–13753, doi:10.1016/j.ijhydene.2017.03.080.
20. Borbáth, I.; Zelenka, K.; Vass, Á.; Pászti, Z.; Szijjártó, G.P.; Sebestyén, Z.; Sáfrán, G.; Tompos, A. CO Tolerant Pt Electrocatalysts for PEM Fuel Cells with Enhanced Stability against Electrocorrosion. *Int. J. Hydrogen Energy* **2021**, *46*, 13534–13547, doi:10.1016/j.ijhydene.2020.08.002.
21. Mohai, M.; László, K.; Bertóti, I. Reduction and Covalent Modification of Graphene-Oxide by Nitrogen in Glow Discharge Plasma. *Surf. Interface Anal.* **2018**, *50*, 1207–1212, doi:10.1002/sia.6411.
22. Naumkin, A. V.; Kraut-Vass, A.; Gaarenstroom, S.W.; Powell, C.J. NIST X-Ray Photoelectron Spectroscopy Database 2023.
